# Myeloid-Derived Suppressor Cells in Bacterial Infections

**DOI:** 10.3389/fcimb.2016.00037

**Published:** 2016-03-31

**Authors:** Michael Ost, Anurag Singh, Andreas Peschel, Roman Mehling, Nikolaus Rieber, Dominik Hartl

**Affiliations:** ^1^Children's Hospital, University of TübingenTübingen, Germany; ^2^Infection Biology Department, Interfaculty Institute of Microbiology and Infection Medicine, University of TübingenTübingen, Germany; ^3^Department of Pediatrics, Kinderklinik München Schwabing, Klinikum Schwabing, StKM GmbH und Klinikum rechts der Isar, Technische Universität MünchenMunich, Germany

**Keywords:** MDSC, myeloid-derived suppressor cells, bacteria, infection, immune suppression, sepsis

## Abstract

Myeloid-derived suppressor cells (MDSCs) comprise monocytic and granulocytic innate immune cells with the capability of suppressing T- and NK-cell responses. While the role of MDSCs has been studied in depth in malignant diseases, the understanding of their regulation and function in infectious disease conditions has just begun to evolve. Here we summarize and discuss the current view how MDSCs participate in bacterial infections and how this knowledge could be exploited for potential future therapeutics.

## Introduction

Bacterial infections represent one of the major threats for the human immune system. Particularly, in vulnerable populations, such as elderly people or patients after surgery, they can lead to sepsis or death (Martin et al., 2003). A functional immune response is a key factor to control the outcome of bacterial infections. Therefore, the human immune system has evolved several effector mechanisms to combat bacteria, involving the innate and the adaptive arm of the immune system. While phagocytic cells, mainly neutrophils and macrophages, are traditionally regarded as key players in host-bacteria interactions (Kruger et al., 2015), research focus has shifted toward a heterogeneous group of myeloid cells, which suppress immune responses, termed myeloid-derived suppressor cells (MDSCs) (Gabrilovich et al., 2007). First described in cancer (Young et al., 1987; Gabrilovich and Nagaraj, 2009; Waldron et al., 2013), subsequent studies highlighted the potential role of MDSCs in auto-immune and infectious diseases (Haile et al., 2008; Tacke et al., 2012). Notably, MDSC induction and immunosuppressive activity has been shown in infections with hepatitis C virus (Tacke et al., 2012; Goh et al., 2016). Elevated MDSCs were also found in HIV patients (Qin et al., 2013; Tumino et al., 2015), in other viral infections as well as in fungal and parasitic infections (Van Ginderachter et al., 2010; Goh et al., 2013; Rieber et al., 2015). Distinct MDSC subphenotypes have been described depending on the infectious agent and the stage of disease (Norris et al., 2013; Janols et al., 2014). Therapeutically, several approaches on how to interfere with or target MDSCs have been discovered and are subject to preclinical and clinical studies in cancer (Gabrilovich et al., 2001; Ko et al., 2009; Nagaraj et al., 2010b). In this review, we describe the state of research on MDSCs in bacterial infections. Furthermore, we focus on the molecular mechanisms that mediate pathogen recognition and MDSC activation in bacterial infections.

## MDSCs

### MDSC characterization

MDSCs comprise a heterogeneous group of immature myeloid cells that suppress effector immune cells, mainly T-cells and natural killer (NK) cells. Two major MDSC subsets have been described that differ substantially by morphology as well as immunosuppressive mechanisms: (i) granulocytic/neutrophilic MDSCs (PMN-MDSCs) and (ii) monocytic MDSCs (M-MDSCs) (Youn et al., 2008). In mice, PMN-MDSCs are CD11b^+^Ly6G^+^Ly6C^low^, whereas M-MDSCs are CD11b^+^Ly6G^−^Ly6C^high^ (Movahedi et al., 2008; Youn et al., 2008). In humans, MDSCs have been described as CD11b^+^CD33^+^HLA-DR^low∕neg^ cells (Almand et al., 2001; Ochoa et al., 2007). The subset of PMN-MDSCs is CD14^−^ and expresses CD66b^+^ and CD15^+^, while M-MDSCs are CD14^+^ (Zea et al., 2005; Filipazzi et al., 2007; Condamine et al., 2015). Divergent gene expression profiles have been proposed to allow discrimination between MDSCs and other granulocytes/monocytes (Gabrilovich et al., 2012; Condamine et al., 2015). However, the phenotypic characterization is not sufficient to identify MDSCs and an additional proof of the immunosuppressive function is necessary. While PMN-MDSCs have been described as the predominant subset in many cancers, M-MDSCs are involved in melanoma (Filipazzi et al., 2007; Mandruzzato et al., 2009) and chronic infections (Cai et al., 2013; Nagaraj et al., 2013). M-MDSCs are also capable of differentiating into PMN-MDSCs (Youn et al., 2013).

### MDSC expansion and activation

**Figure 1:**
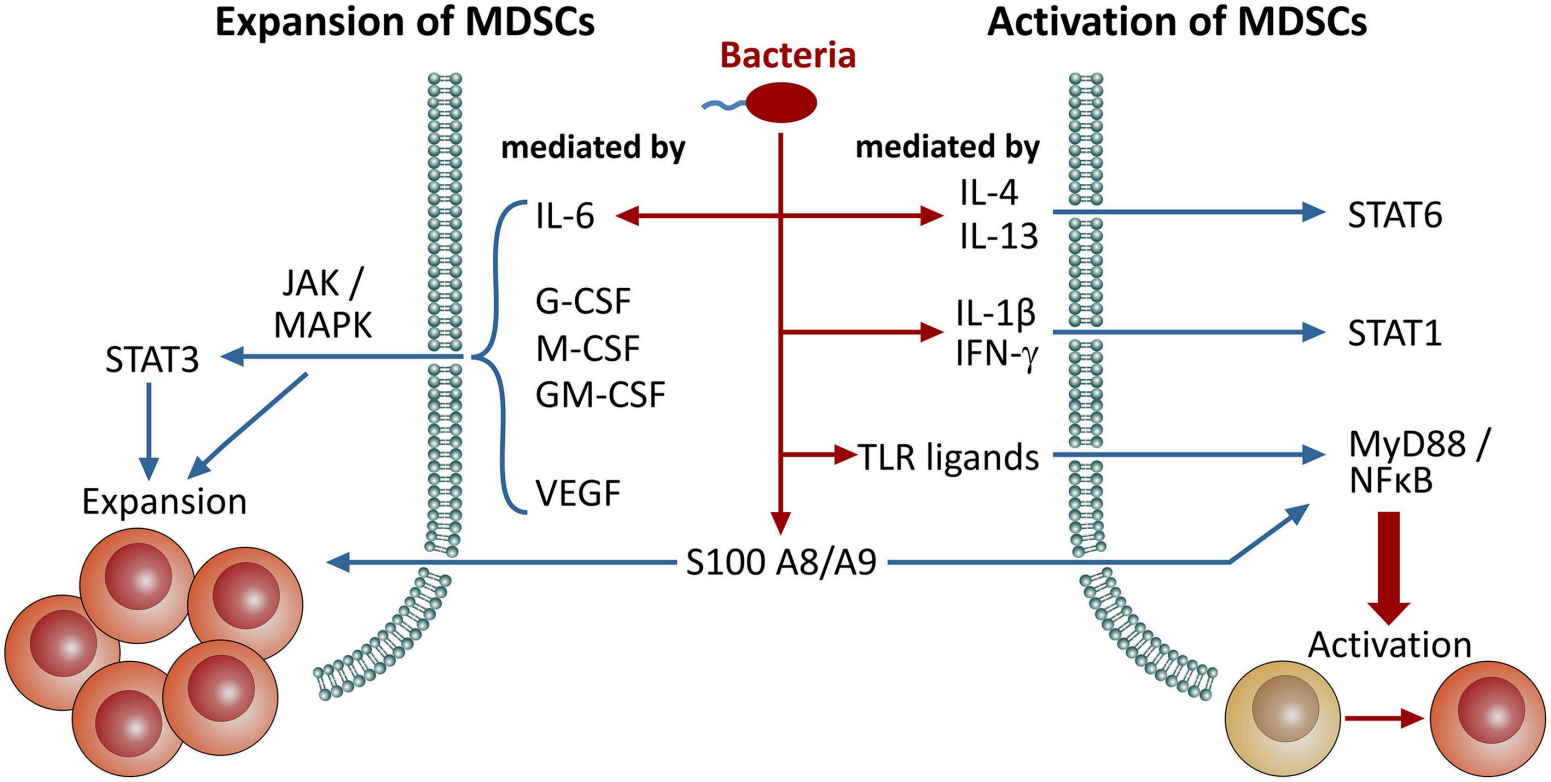
**Signaling pathways involved in the expansion and activation of MDSCs**. Induction/expansion and activation of MDSCs can be triggered through distinct pathways. Here, we provide an overview on different signaling molecules and pathways involved in these events. Bacterial infections either directly promote MDSC activation through microbial patterns (PAMPs), TLR ligation and NFκB-dependent pathways or indirectly through pro-inflammatory mediators, such as several interleukins and IFN-γ, that are secreted upon infection. Furthermore, S100 proteins are also involved in both of these processes.

Immature myeloid cells can be found in healthy individuals at low amounts in peripheral blood (Almand et al., 2001), which increase upon cancer, inflammation and infection. MDSC expansion and activation mechanisms depend on the MDSC phenotype and the species studied (Serafini, 2013; Condamine et al., 2015; Figure 1). MDSC expansion is mainly driven by STAT3, a transcription factor activated by GM-CSF, G-CSF, VEGF as well as IL-6 (Gabrilovich et al., 1998; Serafini et al., 2004; Song et al., 2005; Sawanobori et al., 2008), that influences cell proliferation and differentiation (Yu et al., 2009). Activated STAT3 also induces expression of S100A8 and A9 (Foell et al., 2007), which block differentiation of immature myeloid cells and lead to expansion of MDSCs (Cheng et al., 2008). *In vivo* inhibition of STAT3 via receptor tyrosine kinase inhibitor Sunitinib resulted in a lower amount of MDSCs (Xin et al., 2009). Other related transcription factors of the STAT family, particularly STAT1 and STAT6, also play a role in MDSC activation and function (Movahedi et al., 2008; Munera et al., 2010). STAT1 can be triggered by IFN-γ, whereas STAT6 response is initiated by IL-4 and IL-13 (Rutschman et al., 2001). Downstream, MDSC activation is primarily mediated by NFκB, which is triggered by pro-inflammatory mediators such as IL-1β and TNF-α (Tu et al., 2008; Hu et al., 2014) or toll-like receptor signaling via MyD88 (Delano et al., 2007). Furthermore, NFκB is involved in the ER stress response that is active in MDSCs (Condamine et al., 2014).

### Immunosuppressive mechanisms of MDSCs

MDSCs are employed with several mechanisms to suppress immune cells. MDSCs express arginase-1, an enzyme that converts L-arginine into urea and L-ornithine (Wu and Morris, 1998), which is required for functional T-cell responses (Zea et al., 2004). MDSCs are equipped with another enzyme targeting L-arginine, the inducible NO-synthase (iNOS) that catalyzes the production of citrulline and NO from L-arginine (Wu and Morris, 1998), thereby amplifying L-arginine deprivation. Additionally, NO disrupts signaling pathways downstream of the IL-2 receptor (Mazzoni et al., 2002), promoting T-cell apoptosis (Garban and Bonavida, 2001) and formation of peroxynitrite. This represents one of the most powerful oxidants that is capable of altering the TCR and CD8-molecules via nitration. Thereby these receptors no longer react to antigen-specific stimulation (Nagaraj et al., 2007). Chemokines, such as CCL2, can be nitrated and amino acids as cysteine can be oxidated by peroxynitrite, which impairs T-cell response (Molon et al., 2011). MDSCs also interfere directly with cysteine metabolism by importing cysteine, but lack of an export mechanism contrary to other myeloid cells. As consequence, T-cells run short of cysteine and are left with impaired activation and function (Srivastava et al., 2010). Beyond NO, MDSCs produce another source of oxidants, reactive oxygen species (ROS) (Youn et al., 2008), which disrupt the T-cell function by modifying its TCR-ζ-chain (Nagaraj et al., 2010a). Importantly, MDSC subsets differ in their immunosuppressive mechanisms (Movahedi et al., 2008; Youn et al., 2008). While M-MDSCs and PMN-MDSCs express comparable amounts of arginase-1, substantial differences are found for NO and ROS. M-MDSCs mainly generate NO (Movahedi et al., 2008), whereas PMN-MDSCs produce higher levels of ROS (Youn et al., 2008). Beyond suppressing T-cells, MDSCs also interact in a more dynamic way with T-cells by acting as antigen presenting cells for CD8^+^ T-cells (Watanabe et al., 2008). Additionally, MDSC activity is enhanced by activated T-cells (Nagaraj et al., 2012), while T-cells can also induce MDSC apoptosis by engaging the Fas/FasL axis (Sinha et al., 2011). Besides dampening T-cells, MDSCs are also known to influence the activity and function of other myeloid cells (Ostrand-Rosenberg et al., 2012). By releasing IL-10, MDSCs suppress IL-12 production by macrophages and DCs, rendering them less capable of activating T-cells (Sinha et al., 2007). Another subset of cells dampening T-cell responses are regulatory T-cells (T^reg^), which exhibit cross-talk with MDSCs (Hoechst et al., 2008). MDSCs have been shown to promote the expansion of T^regs^ (Hoechst et al., 2008; Serafini et al., 2008), while some other studies demonstrate more complex scenarios of interaction (Dugast et al., 2008; Movahedi et al., 2008).

## MDSCs and bacterial infections

### TLR ligands

Bacterial pathogens are recognized by immune cells through defined pattern recognition receptors (PRRs). These PRRs are capable of identifying so called pathogen-associated molecular patterns (PAMPs) (Janeway and Medzhitov, 2002), typically microbial cell envelope components, nucleic acids, or polysaccharides (Akira et al., 2006). Toll-like receptors (TLRs) represent the prototypic PRRs sensing bacterial infections. TLRs on the cell surface mainly recognize bacterial molecular patterns, while viral pathogens are detected by intracellular TLRs (Kawai and Akira, 2010). TLR2 is a key TLR in bacterial sensing that forms heterodimers with TLR1 and TLR6 (Akira et al., 2006). The TLR1-TLR2 heterodimer binds with lipopeptides of Gram-negative bacteria (Wyllie et al., 2000), whereas lipoproteins of Gram-positive bacteria are recognized by the TLR2-TLR6 heterodimer (Ozinsky et al., 2000). TLR4 responds to bacterial lipopolysaccharides (LPS) (Poltorak et al., 1998), which is localized in the cell membrane of Gram-negative bacteria. Flagellin, a prominent component of bacterial flagella known to stimulate host defense, is detected by TLR5 (Hayashi et al., 2001) and bacterial DNA motifs are sensed by TLR9 (Hemmi et al., 2000). Notably, TLRs can also be activated by molecular patterns that are released from stressed or damaged cells, so called damage- or danger-associated molecular patterns (DAMPs) (Asea et al., 2002). The synthetic lipopeptide and TLR2/6 agonist Pam2CSK4 has been shown to induce MDSC expansion and prolonged MDSC survival (Maruyama et al., 2015). Likewise for TLR4, LPS triggered MDSC expansion and activation using the MyD88-dependent signaling pathway in several *in vitro* as well as *in vivo* studies (Delano et al., 2007; Bunt et al., 2009). While MDSC generation was partly independent of MyD88, MyD88 activity was essential for their immunosuppressive functionality (Hong et al., 2013). We reported previously that the TLR5 ligand flagellin induced MDSC expansion (Rieber et al., 2013). Thus, several TLRs that detect bacterial PAMPs are reported to enhance MDSC frequency and activity. However, some TLR agonists are also used in anti-tumor therapy and show adverse effects on MDSC expansion and activity (Aranda et al., 2014). Hence, studies reported that Poly (I:C), a TLR3 agonist, reduced MDSC frequency and inhibited immunosuppressive effects (Zoglmeier et al., 2011). Stimulation of TLR9 with CpG oligonucleotides induced differentiation of M-MDSCs and led to a loss of their immunosuppressive function (Zoglmeier et al., 2011; Shirota et al., 2012). A combination of TLR7-9 ligands enhanced anti-tumor responses by NK cells and cytotoxic T-cells and reduced MDSC frequency (Zhao et al., 2014).

### Bacteria

Several Gram-positive and -negative bacteria have been shown to induce or modulate MDSCs *in vitro* and *in vivo*. These studies are summarized and discussed in the section below (Table 1).

**Table 1:** **Bacterial MDSC induction and impact on disease outcome**.

**Pathogen**	**Expanding MDSC subsets**	**Study type**	**Outcome**	**References**
*Staphylococcus aureus*	PMN- and M-MDSCs	*In vitro, in vivo* (mouse and human)	Aggravation of infection	Thurlow et al., 2011; Heim et al., 2014, 2015a,b; Skabytska et al., 2014; Tebartz et al., 2015
*Mycobacterium tuberculosis*	PMN- and M-MDSCs	*In vivo* (mouse and human)	Aggravation of infection	Obregon-Henao et al., 2013; du Plessis et al., 2013; Knaul et al., 2014; Tsiganov et al., 2014; Yang et al., 2014; El Daker et al., 2015
*Pseudomonas aeruginosa*	PMN-MDSCs	*In vitro, in vivo* (human)	Host protection (associated with better lung function)	Rieber et al., 2013
*Klebsiella pneumoniae*	PMN-MDSCs	*In vivo* (mouse)	Host protection	Cai et al., 2009; Poe et al., 2013
*Porphyromonas gingivalis*	*Not mentioned*	*In vivo* (mouse)	*Not mentioned*	Ezernitchi et al., 2006
Polymicrobial sepsis	PMN- and M-MDSCs	*In vivo* (mouse and human)	Host protection	Delano et al., 2007; Sander et al., 2010; Brudecki et al., 2012; Darcy et al., 2014; Janols et al., 2014; McClure et al., 2014

*Staphylococcus aureus* is a Gram-positive bacterium and a major bacterial pathogen in humans that mainly colonizes the nasal cavity of 20–30% of the population and poses a risk of invasive infections for these carriers (Foster, 2004; Weidenmaier et al., 2012). Antibiotic-resistant strains, particularly methicillin-resistant *S. aureus* (*MRSA*) represent a major problem all over the world (Smith et al., 1999; Saeed et al., 2014). Lipoproteins anchored to the cytoplasmic membrane are known to act as TLR2-ligands (Nguyen et al., 2015) and it is already known that *S. aureus* is able to evade immune responses by impairing T-cell function (Fedtke et al., 2004; Schreiner et al., 2013). The expansion of both MDSC subsets and immunosuppressive activity was shown in *S. aureus* skin infection models (Skabytska et al., 2014). MDSC-mediated immune suppression was mainly dependent on iNOS and, to a lesser extent, on arginase-1. *S. aureus* causes infections and forms biofilms in orthopedic implants where an impaired immune response has been reported (Thurlow et al., 2011). In these biofilms, elevated MDSC frequencies have been found with enhanced expression of arginase-1, iNOS and IL-10 (Heim et al., 2014). Consistent with these findings, depletion of MDSCs led to improved bacterial clearance (Heim et al., 2014), while MDSC activity increased disease severity in this biofilm model (Heim et al., 2015b). In line with this concept, it was shown that adoptive transfer of MDSCs in *S. aureus* infected mice led to an aggravation of disease (Tebartz et al., 2015). Taken together, the studies on MDSCs in *S. aureus* infections suggest that MDSCs play a rather harmful role in *S. aureus* infected hosts.

Tuberculosis due to infection with *Mycobacterium tuberculosis* is one of the most prominent infectious diseases worldwide with an estimated 9 million reported cases annually and reports suggest that mortality rates are much higher than those of other bacterial infections (Jassal and Bishai, 2010). In mice, heat-killed *M. tuberculosis* is able to induce MDSCs, which produce NO and superoxide anion (Dietlin et al., 2007). Likewise, patients with active tuberculosis as well as patients that had been recently exposed with *M. tuberculosis* exhibited expanded MDSC frequencies in their peripheral blood and bronchoalveolar lavage samples (du Plessis et al., 2013; Yang et al., 2014; El Daker et al., 2015). *In vivo* studies further demonstrated that MDSCs accumulated in lungs of infected mice where they phagocytized but did not kill the mycobacteria, thereby providing a shelter for intracellular bacteria survival (Knaul et al., 2014). Depletion of MDSCs led to an increase of T-cell frequencies, reduced bacterial burden and improved disease pathology (Knaul et al., 2014), while accumulation of MDSCs was linked with progress and severity of tuberculosis (Tsiganov et al., 2014). However, in a different study phenotypical MDSC-like cells were induced by *M. tuberculosis* but failed to inhibit T-cell proliferation. These cells rather promoted Th17 responses (Obregon-Henao et al., 2013), which is in line with previous reports on MDSC-Th17 interactions (Yi et al., 2012; Zhang et al., 2015). Attenuated *Mycobacterium bovis*, which is also partly used for vaccination against TB, leads to MDSC expansion in a MyD88-dependent manner (Martino et al., 2010). In a similar setting, two subsets of MDSC-like cells were generated recently. M-MDSCs acted as expected, however phenotypical copies of PMN-MDSCs lacked immunosuppressive activity, and rather enhanced the proliferation of CD4^+^ and CD8^+^ T-cells (Zhan et al., 2015).

*Pseudomonas aeruginosa*, a flagellated, partly opportunistic and gram-negative bacterium is mainly recognized by the immune system through flagellin/TLR5 signaling (Zhang et al., 2005) and other sensors such as NLRC4 (Franchi et al., 2007). Infections with *P. aeruginosa* are known to affect especially vulnerable patients in a hospital-acquired manner, most frequently ventilated patients in intensive care units, patients with severe burns, cystic fibrosis (CF) patients and chronic obstructive pulmonary disease (COPD) patients. Bacterial clearance by the immune system of these vulnerable patients is often not successful (Cohen and Prince, 2012). We demonstrated previously that CF patients with chronic *P. aeruginosa* infections featured a higher MDSC frequency in their peripheral blood compared to CF patients without *P. aeruginosa* infections or healthy control subjects (Rieber et al., 2013). In *P. aeruginosa*-infected patients, the percentages of MDSCs correlated with pulmonary function (Rieber et al., 2013). This suggests that MDSC activity induced by *P. aeruginosa* prevents excessive inflammation and leads to improved lung function. As the MDSC expansion was dependent on flagellin as TLR5 ligand, further flagellated bacteria such as *Helicobacter pylori* or flagellated *Escherichia coli* strains may also induce MDSC accumulation in the same manner. An increase in MDSC frequency in *H. pylori* infected mice and humans has already been reported (Zhuang et al., 2015). Since there were no studies done using flagellin-deficient *H. pylori*, the potential role of flagellin in this MDSC expansion setting remains elusive.

*Klebsiella pneumoniae* is another cause of severe pneumonia, mostly acquired in hospitals (Jones, 2010). It is known to activate TLR2 and TLR4 signaling during the infection (Wieland et al., 2011). In mice, infection with *K. pneumoniae* promoted MDSC expansion and thus increased levels of IL-10 (Poe et al., 2013). IL-10 deficient mice were able to clear the infection, but had persistent lung inflammation and enhanced morbidity after infection (Poe et al., 2013). Consequently, IL-10 dependent MDSC activities seem to play a key role in *K. pneumoniae* infection recovery. *K. pneumoniae* infected mice further showed decreased bacterial clearance as well as reduced survival when MyD88 was knocked out (Cai et al., 2009). Contrary to the latter studies, another study found no evidence for MDSC expansion in peripheral blood of pneumonia patients compared to healthy controls (Zhang et al., 2013). However, pneumonia patients included patients with RSV and Rhinovirus infections, so no clear conclusions for bacterial lung infections can be drawn from this study.

Sepsis is defined as a bloodstream infection with a systemic inflammatory response-syndrome (Levy et al., 2003). The most common bacteria in bloodstream infections are *S. aureus, E. coli*, coagulase-negative *Staphylococci* and *K. pneumonia* (Weinstein et al., 1997). MDSC expansion and activity during sepsis has been reported in several studies. In a model of polymicrobial sepsis, a MyD88-dependent MDSC expansion was immunosuppressive mainly against CD8^+^ T-cells (Delano et al., 2007). In a similar model the transfer of MDSCs 10 days after induction also inhibited T-cell proliferation and improved the survival rate of septic mice (Derive et al., 2012). Though 3-day-old MDSCs were still able to suppress T-cell proliferation, they expressed less immunosuppressive enzymes after LPS-stimulation and did not improve survival (Derive et al., 2012). The beneficial role of MDSCs in sepsis was supported by another study, which showed that hepatic acute phase proteins were essential for MDSC induction in polymicrobial sepsis and MDSCs prevented sepsis-associated mortality (Sander et al., 2010). In line with murine studies, MDSC expansion and immunosuppressive activity with enhanced expression of arginase was also found in patients with sepsis (Darcy et al., 2014). The induction of MDSCs in sepsis has been linked to specific microRNA signatures (McClure et al., 2014). While PMN-MDSCs were primarily found in sepsis patients with Gram-positive pathogens, M-MDSCs expanded regardless of the Gram staining in all sepsis patients (Janols et al., 2014). Thus, in contrast to the findings in the *S. aureus* orthopedic implant infection model where MDSCs were harmful, in sepsis MDSCs seem to act in favor of the host. The underlying mechanism for this discrepancy remains to be dissected in future studies, but could be due to (i) the infected compartment (systemic/sepsis vs. localized/compartmentalized) and/or (ii) the respective bacterial pathogen(s).

The Gram-negative *Porphyromonas gingivalis* is an anaerobic bacterium that is mainly found in the oral cavity where it causes periodontal disease. Mice infected with this bacterium showed an accumulation of MDSCs in their spleen and elevated MDSC frequency in the peripheral blood (Ezernitchi et al., 2006). In mice with chronic *Porphyromonas* infection, T-cell function was impaired by modulation of the TCR-ζ-chain (Ezernitchi et al., 2006).

### Infection-associated mediators

Bacterial infections induce the production of a plethora of pro-inflammatory cytokines and chemokines. Many of them have also been linked to MDSC expansion and activation in addition to their boost of immune responses against bacteria. Hereby, Interleukins are of great importance, mainly e.g., IL-1β, IL-4 and IL-6. IL-1β is known to promote MDSC accumulation and suppress T-cell responses (Song et al., 2005). Consistently, blocking IL-1 receptor signaling inhibits MDSC function (Tu et al., 2008). An explanation could be that IL-1β is known to enhance NO production by triggering iNOS expression (Kanno et al., 1994; Kwon et al., 1995), which has been reported to mediate immunosuppression by MDSCs. Similarly, IL-4 and IL-13 trigger arginase-1 expression (Rutschman et al., 2001). IL-4 is mainly produced by activated Th2 cells during inflammation (Bronte et al., 2003) and the IL-4 receptor IL-4Rα was found to be upregulated on MDSCs (Mandruzzato et al., 2009). Blockade of IL4Rα has been reported to induce MDSC apoptosis (Roth et al., 2012). Furthermore, IL-6 not only induces MDSC expansion (Garg and Spector, 2014), but also stimulates the production of ROS as well as arginase-1 (Chen et al., 2014). Blocking IL-6 led to reduced STAT3 signaling (Wu et al., 2012). In addition to the aforementioned cytokines, TNF-α has also been reported to promote both MDSC expansion and survival (Zhao et al., 2012). Signaling via TNFR-2 leads to NFκB activity, and thereby amplifies immunosuppressive mechanisms of MDSCs (Hu et al., 2014). MDSC expansion is enhanced by inhibiting myeloid cell differentiation, an effect mediated e.g., by S100A8 and S100A9 in a STAT3-dependent manner (Cheng et al., 2008). These S100 proteins were also found to be secreted by MDSCs (Sade-Feldman et al., 2013) generating an autocrine feedback loop (Sinha et al., 2008). Notably, S100A8 and S100A9 not only lead to inhibition of myeloid differentiation, but also attract MDSCs to sites of inflammation via NFκB (Sinha et al., 2008). Generated peptide-FC fusion bodies, so called peptibodies, target S100A8 and S100A9 and were able to deplete MDSCs *in vitro* as well as *in vivo* (Qin et al., 2014).

## Conclusions

While cancer-associated MDSCs are traditionally in the focus of research, attention has recently shifted toward the potential role of MDSCs in bacterial infections. MDSC expansion can be triggered through PAMPs from Gram-positive and Gram-negative bacteria (Delano et al., 2007; Maruyama et al., 2015). It has been further proposed that PMN-MDSCs mainly expand in infections caused by Gram-positive bacteria, while M-MDSCs were induced regardless of the Gram staining (Janols et al., 2014). Yet, some principles of TLR-induced MDSC generation remain unclear. While downstream signaling of TLR4 and TLR9 both merge on the MyD88-dependent pathway, TLR4 was found to mediate MDSC expansion and activation, while TLR9 led to reduced MDSC frequencies (Delano et al., 2007; Zoglmeier et al., 2011). Particularly, *S. aureus* and *M. tuberculosis* have been shown to potently induce MDSC expansion and MDSCs aggravated disease severity *in vivo* (Tsiganov et al., 2014; Tebartz et al., 2015). However, in other infectious disease conditions, MDSCs were associated with an improved outcome, such as *P. aeruginosa* infections in CF patients (Rieber et al., 2013) or in polymicrobial sepsis (Sander et al., 2010). The future challenge remains how to translate these findings into therapeutic approaches. A potential therapeutic strategy is to target/deplete MDSCs in settings where they seem to do more harm than good (*S. aureus* orthopedic implant infections and *M. tuberculosis* infections). Pharmacologically, the tyrosine-kinase inhibitors Sunitinib and Sorafenib were shown to interfere with STAT3 signaling and to effectively reduce MDSC populations (Ko et al., 2009; Cao et al., 2011). A similar effect can be achieved by using all-trans-retinoic acid (ATRA), an active metabolite of vitamin A (Almand et al., 2001; Mirza et al., 2006). Furthermore, the chemotherapeutic agents 5-Fluoruracil and gemcitabine have been shown to selectively eliminate MDSCs (Suzuki et al., 2005; Vincent et al., 2010). Conversely, *in vivo* expansion or adoptive transfer of MDSCs represents a promising strategy in *P. aeruginosa* infections or sepsis.

## Author contributions

MO searched databases and literature, wrote the manuscript, discussed data, composed the figure and created the table. AS contributed to manuscript writing. AP contributed to the S. aureus manuscript chapter writing and discussion part. RM contributed to manuscript writing. NR contributed to discussion of the literature and manuscript writing. DH co-wrote the manuscript, discussed findings and contributed to figure design.

## Funding

We thank the IZKF, University of Tübingen, the Deutsches Zentrum für Infektionsforschung (DZIF) and the DFG SFB/CRC685, University of Tübingen, for financial support.

### Conflict of interest statement

The authors declare that the research was conducted in the absence of any commercial or financial relationships that could be construed as a potential conflict of interest.
